# HIV, HPV and *Chlamydia trachomatis*: impacts on male fertility

**DOI:** 10.5935/1518-0557.20200020

**Published:** 2020

**Authors:** Ana Carolina Xavier Goulart, Hana Carolina Moreira Farnezi, Juliana Peralva Baumgratz Medeiros França, Adriana dos Santos, Mariana Gontijo Ramos, Maria Lectícia Firpe Penna

**Affiliations:** 1 Faculdade de Ciências Humanas, Universidade FUMEC, Belo Horizonte, MG, Brazil

**Keywords:** HIV, HPV, *Chlamydia trachomatis*, male infertility, semen analysis

## Abstract

Sexually transmitted diseases (STDs) are pathologies that have viruses, bacteria, protozoa and fungi as infectious agents, affecting millions of people worldwide and causing physical and psychological consequences for the carrier. Some of these infections such as HIV, HPV and *Chlamydia trachomatis* may present an asymptomatic phase, making the diagnosis difficult, which is often only performed when the couple looks for infertility treatment after not achieving spontaneous pregnancy. Infertility affects 15% of couples, 50% of cases are male-related, and it is estimated that STDs, which interfere with the physiology of the male reproductive system and may impair semen in parameters such as motility, concentration, morphology and number, cause 15% of male infertility cases. Since STDs treatments are increasing the expectation and quality of life of infected patients, discussing issues such as sexuality and reproduction is of great importance in clarifying unknown facts. This paper aims to discuss how the infectious processes associated with HIV, HPV and *Chlamydia trachomatis* can interfere with semen quality causing male infertility without apparent cause.

## INTRODUCTION

The World Health Organization (WHO) defines infertility as “the inability of a sexually active, non-contraceptive couple to achieve pregnancy in one year”. The problem affects 15% of couples and 50% of cases are related to men (Cui, 2010). Since the 1930s, studies carried out in several countries have shown that the average quality of the semen has been decreasing worldwide. There is no conclusive information on the causes; the main suspects are alcohol, cigarette smoking and chemicals in pesticides, solvents and plastic containers.

The functioning of the male reproductive system depends on hormonal regulation from the hypothalamus - pituitary - gonadal axis, in order to achieve complete spermatogenesis, storage and transport of spermatozoa. Some sexually transmitted diseases (STDs) may interfere in this physiology, causing infertility (Fode *et al*., 2016). It is estimated that STDs cause 15% of male infertility cases (Pellati et al., 2008).

STDs are responsible for some acute illnesses, infertility, severe physical and psychological consequences, and in some cases can lead to death (Geremew *et al*., 2017). According to the WHO, there are more than 30 species of bacteria, viruses and parasites capable of being transmitted through sexual contact, and every day more than 1 million people are infected (WHO, 2019). Among the STDs associated with infertility are human immunodeficiency virus (HIV), human papillomavirus (HPV) and *Chlamydia trachomatis* (CT), which can present asymptomatic phases, making it difficult to diagnose these pathologies early on (Mendonça & Netto, 2005; Loreto & Azevedo-Pereira, 2018; Brazil, 2015).

In Brazil, from 2007 to 2018, 247.795 cases of HIV infection were reported, of which 169,932 (68.6%) were men. By February of 2019, there were 233 cases reported (Brazil, 2018). An estimated 1.2 million people are living with HIV in the United States. Of these individuals, approximately 86% are in reproductive age (15 to 44 years) (Hanson & Dorais, 2017). Studies show that HIV prevalence in South Africa is among the highest in the world; about 18.8% of the population, aged 15-49 is infected with the virus (van Zyl & Visser, 2015).

HPV in men is little discussed because most infections are benign, being associated with interference in seminal parameters and a higher rate of abortion in case of fertilization in assisted reproduction clinics (Yang *et al*., 2013). HPV is not a notifiable disease, but a survey carried out in 2017 with Brazilian, American and Mexican men between the ages of 18 and 70 indicated that approximately 72% of Brazilian men have the infection (Sudenga *et al*., 2016).

CT infection is not of mandatory notification in Brazil, according to the country’s Ministry of Health; however, the WHO estimates that 131 million people are infected annually. The difficulty in finding an accurate estimate in these cases is also because the disease is asymptomatic and difficult to diagnose (Brazil, 2015).

The objective of this study is to investigate the relationship between human immunodeficiency virus (HIV), Human papillomavirus (HPV) and *Chlamydia trachomatis* and male infertility.

## METHODS

We carried out an integrative review using PubMed with STDs and male infertility as keywords. The search was restricted to the English language and to studies in humans, between 1993 and 2019, using the terms "HIV and male infertility", “HPV and male infertility" and “*Chlamydia trachomatis* and male infertility". We had 112 papers initially and, after the analysis, 28 were chosen for the present study. Papers in reverse search were considered in the making of this study.

## RESULTS

### Human immunodeficiency virus

The sperm abnormalities found in HIV patients are poorly understood, since both the virus and the antiretroviral treatment can cause the changes (La Vignera *et al*., 2011; Waters*et al*., 2007). Men with HIV had increased viscosity (Ochsendorf, 2008), decreased ejaculate volume and lower sperm motility. The hypothesis to explain decreased motility is related to mitochondrial toxicity caused by the nucleotide reverse transcriptase inhibitors (NRTIs) used in therapy. This happens due to the decrease in ATP production, which is a fundamental molecule in spermatozoa movement (Frapsauce *et al*., 2015; Wang *et al*., 2014). The decrease in volume and increase in semen viscosity may be associated with the functional alterations in the seminal vesicles and prostate (Ochsendorf, 2008).

Frapsauce *et al*. (2015) divided 378 HIV-infected men into four groups: a) in the first group, 66 patients who had not undergone any type of treatment; b) the second group had 49 patients who received only NRTI inhibitors; c) in the third group, 144 patients received NRTIs + protease inhibitor (PI) and finally, d) in the fourth group, 119 patients received non nucleotide reverse transcriptase inhibitors (NNRTIs) + NRTIs. Semen analysis was based on guidelines from the World Health Organization (WHO) of 1999. Sperm motility differed significantly between the groups, the percentage of fast and progressive spermatozoa (type A) in the group receiving NNRTI regimen was half the value found in the other three groups. There were no differences in sperm motility between patients who received no treatment and those who received only NRTIs or NRTIs + IP. Other parameters such as pH, viscosity, vitality, volume and morphology did not change significantly.

La Vignera *et al*. (2011) state that the effects of HIV infection on sperm parameters can be seen in both asymptomatic and symptomatic patients. All men with AIDS had abnormal leukospermia and spermatozoa, in addition to reports of a larger quantity of spermatozoa with residual cytoplasm and immature germ cells. Those with HIV-1 had significantly lower ejaculate volume, but sperm concentration, total count, progressive motility and morphology were normal in comparison to controls. The TUNEL analysis revealed an increase in the percentage of spermatozoa ejaculated with DNA fragmentation in the semen.

The CD4 lymphocyte count serves as a laboratory indicator for assessing the immunity of HIV patients, those with less than 350 cells/µl are more susceptible to opportunistic infections (Wang *et al*., 2014). In the study by Wang *et al*. (2014) the semen of 33 HIV-infected men was analyzed and the results showed that sperm vitality, sperm motility (A + B), total sperm motility and sperm penetration rates were significantly higher in patients with the CD4 count greater than 350 cells/µl compared to those with CD4 counts less than 350 cells/µl. In addition, this study also demonstrated that patients who received antiretroviral therapy did not present significant differences in semen parameters, such as spermatozoa volume, motility, vitality and morphology. However, they showed an improvement in the sperm penetration rate.

A study carried out by Umapathy (2005) in Zimbabwe with 83 patients showed that 36 were HIV-positive and 47 HIV-negative. After the semen analysis, they concluded that seropositive men have less mobile spermatozoa and leukospermia when compared to their seronegative counterparts, since the total sperm counts, seminal volume and sperm morphology did not present statistically significant alterations. Leukospermia was the most remarkable parameter, making it possible to associate it with impaired sperm motility. This can be explained by the activation of leukocytes, which can cause oxidative stress in spermatozoa.

Bujan *et al*. (2007) did not associate detectable viral load with changes in sperm parameters; however, when the HIV genome is present in semen cells, there is an increase in the number of polymorphonuclear cells, a decrease in total sperm count and on motility. There was no relationship between sperm alterations, duration and type of treatment performed among seropositive patients.

Rusz *et al*. (2012) also described the increase of immature cells and residual cytoplasm in the semen of HIV-positive men, whereas AIDS patients had leukospermia, abnormal spermatozoa and testicular atrophy. Data on the impact of HIV on fertility are important, since antiretroviral treatment increases patients’ life expectance and quality, so sexuality and procreation issues are of great relevance (Rusz *et al*., 2012).

### Human papillomavirus

HPV is a microorganism that causes one of the most common STDs in the world (Luttmer *et al*., 2016). Its main clinical consequence is cervical cancer, being the main cancer-related cause of death among young women (Gizzo *et al*., 2014). Due to lack of symptoms, the infection was considered of no clinical importance for men, so few studies were done in the area (Garolla *et al*., 2013). It is estimated that 38.5 per 1000 men are infected with HPV per month and some studies have been trying to demonstrate if there is a relationship between infection and male infertility (Gizzo *et al*., 2014).

HPV DNA and RNA have already been identified in the penis, glans, urethra, epididymis, testis, semen, and exfoliative cells (Garolla *et al*., 2012). Biological evidence suggests the presence of the virus in two regions of the equatorial end of the spermatozoa head, and it can cause damage to semen quality (Lyu *et al*., 2017). In an analysis of the interaction between the viral capsid protein L1 and the syndecan-1 in spermatozoa, immunofluorescence tests showed that the virus is present in the head, but it is not bound to the acrosome, suggesting that HPV infects spermatozoa by primary binding with syndecan-1 (Garolla *et al*., 2012).

In addition to the seminal parameters, Garolla *et al*. (2013) investigated the association between the HPV infection in semen and antisperm antibodies (ASA) in a group of 257 men (61 infertile HPV positive, 104 infertile HPV negative and 92 fertile controls). Among the infected patients there was a decrease in progressive sperm motility and higher rates of ASA on the sperm surface, with 34.7% infected and 15.8% uninfected. This finding suggests the HPV DNA on the surface of the spermatozoa may represent a stimulus for ASA formation. After 12 months the 61 patients were analyzed again, 39 of them remained positive for HPV and 17 presented ASA, while 22 did not present the infection in the semen anymore. There was another examination after 24 months, where 9 patients continued to present the infection and 5 had ASA, while the other 30 patients did not present the infection.

From a study involving 1138 men in China, patients from a fertility clinic and normal controls, Yang *et al*. (2013) reported that among the patients undergoing reproductive treatment (615), 17.4% had the infection, while among the fertile controls (523) 6.7% had HPV. Among the infected patients, there was a loss in motility, vitality and morphology when compared to those not infected. The principle virus genotypes found among patients were HPV-45, -16, -52, -18/59 and -33 (all high-risk), and among controls HPV-81 (low-risk), -68, -33 and -39 (high-risk). Damke *et al*. (2017) studied 229 couples with infertility, and found that 16.6% had the infection, 5.7% being of high risk and 6.1% of low risk. Among HPV positive patients, there was hypospermia, altered viscosity, high pH and a high number of leukocytes, especially among those with an infection of more than one genotype.

In a study, involving 308 male patients from an infertility clinic, 24 (7.8%) had a positive HPV result, most of the infections were of high risk and HPV-52 (21%) was the main genotype found. However, there were no significant changes in seminal parameters (Yang *et al*., 2013). In another study with 299 infertile men, 45.5% had the virus on the surface of the penis, the main genotypes were the HPV-CP6108 and HPV-84 (low-risk), and 13.61% had HPV-53 and HPVCP6108 (low-risk) in the semen sample as the most common. There was no statistical difference between the seminal parameters of men positive and negative for infection (Golob *et al*., 2014).

Luttmer *et al*. (2016) also found a high prevalence of HPV among fertility clinic patients, and HPV16 (23.4%) (High-risk) was the main genotype found in this study with 430 patients, but there was no significant change in relation to uninfected patients. Cortés-Gutiérrez *et al*. (2017) compared samples from 38 men (22 male normozoospermic infertile men, 9 fertile donors and 7 men with a risk factor for HPV infection) and demonstrated that despite the high infection rate among infertile men, there is no increase in DNA fragmentation spermatozoa in HPV positive patients.

### Chlamydia trachomatis

*CT* is related to male infertility because it is a major cause of pelvic inflammation in the reproductive system organs, being responsible for 40 to 80% of epididymitis and, consequently, causing orchitis and prostatitis. Thus, this infection can damage the canalicular system, causing testicular atrophy and obstructive azoospermia. The epididymis acts on sperm maturation and when infected it can affect sperm function (Greendale *et al*., 1993; Stephens *et al*., 2011; Gallegos *et al*., 2008). This process may be due to the presence of inflammatory cells, alterations in the epithelium and in mucus composition (Paavonen & Eggert-Kruse, 1999).

According to Sonnenberg *et al*. (2013), Chlamydia infection causes direct damage to sperm, leading to decreased motility, increased non-viable forms of spermatozoa, and increased lipid peroxidation of cell membranes due to elevated IgA levels. The authors also demonstrated that this infection could trigger apoptosis in sperm cells, leading to DNA fragmentation.

A study in Iran compared 165 clinically asymptomatic infertile men with abnormal seminal parameters, with a control group of 165 fertile men with normal seminal parameters. Of the 165 patients considered infertile, seven were positive for *CT*, whereas in the control group there was one individual with a positive result for the bacterium. Seminal parameters of both groups were compared and there was a statistically significant difference in concentration (millions/ml), total count, progressive motility A, progressive motility B, total progressive motility (A + B + C) and morphology (WHO, 2010). Patients positive for the bacteria were treated with doxycycline for 7 days and after 30 days, the semen analysis was repeated. Thus, there was a statistically significant improvement (> 57%) in almost all parameters, mainly in the concentration and total progressive motility of spermatozoa (A + B + C). There was an exception in the progressive motile class A, which had only a 42.9% improvement. There were no statistically significant differences in volume, pH, viscosity, and non-progressive motility (class C) in the initial and post-treatment analyzes (Ahmadi *et al.*, 2018).

Some studies showed that bacterial infection may harm semen parameters (Bezold *et al*., 2007; Eley *et al*., 2005; Joki-Korpela *et al*., 2009) and cause DNA fragmentation (Gallegos *et al*., 2008). However, others reported that CT does not alter seminal parameters (Günyeli *et al.*, 2011; Hosseinzadeh *et al*., 2004).

In a study by Eggert-Kruse *et al*. (1997), serological tests of 1,303 couples were carried out, showing that men with anti-*CT* antibodies did not differ in semen parameters or ASA levels compared to men without anti-*CT* antibodies, showing that previous CT exposure did not cause male infertility.

## CONCLUSION

STDs may be responsible for changes in seminal quality and may be a possible explanation for several cases of infertility with no apparent cause (Table 1). HIV infection can be related to changes in the parameters of motility, morphology and leukospermia. Despite the observed relationship between the impact of antiretroviral agents and mitochondrial function, we need further studies to determine possible treatment losses. Positive diagnoses for HPV are frequent among patients in assisted human reproduction services; however, the relationship between HPV and infertility remains undetermined. Evidence suggests the presence of viral DNA bound to the spermatozoa head, leading to high ASA concentration, impairment of motility, vitality and morphology, also associated with the infection. There may be changes in concentration, motility and morphology in individuals with *CT* infections. Although antibiotic treatment is effective, we still need more studies with longer duration and larger samples to suggest results that are more consistent.

**Table 1:** Potential damage in the seminal parameters caused by STDs

	AUTOR	POTENTIAL DAMAGE IN THE SEMINAL PARAMETERS
**HIV**	Hanson BM, Dorais JA (2017), van Zyl C, Visser MJ (2015)	No statistical difference was found.
	Frapsauce C et al. (2015)	Sperm motility was the only semen parameter that varied significantly according to treatment status.
	Wang D et al. (2014)	Sperm vitality, sperm mobility (a + b), total sperm mobility and penetration rates were higher in patients with CD4 + > 350 / µl
	Rusz A et al. (2012)	Ejaculate volume, sperm motility, sperm concentration or normal sperm morphology was significantly correlated with the number of CD4+ blood cells
	La Vignera S et al. (2011)	All men with AIDS had abnormal leukocytosemia and sperm, and HIV-positive men had a significantly higher percentage of sperm with cytoplasmic droplets, immature germ cells and sperm cells.
	Ochsendorf FR (2008)	Semen parameters were within normal range in HIV-positive men without symptoms, normal sperm morphology was impaired with disease progression. In patients with AIDS, abnormal sperm and leukocytpermia have been reported.
	Waters L et al (2007)	In men with advanced HIV infection, low testosterone levels are common. HIV is also associated with reduced semen volume and motility.
	Bujan L et al. (2007)	Demonstrate changes in sperm motility and ejaculate volume in HIV-1 infected patients, most of whom were receiving antiretroviral therapy.
	Umapathy E (2005)	Impaired sperm motility in HIV+ men may be mediated by activated seminal leukocytes, which may induce oxidative stress on sperm. Leukocytospermia may be a reliable indicator of HIV+
	Fode M et al. (2016)	The authors concluded HIV+ men with low CD4 + cell counts or severe symptoms described reductions in semen quality
**HPV**	Lyu et al. (2017)	Suggests the presence of the virus in two regions of the equatorial end of the spermatozoa head, may cause damage to semen quality.
	Damke et al. (2017)	Hypospermia, altered viscosity, high pH and a high number of leukocytes.
	Cortés-Gutiérrez et al. (2016)	Ddespite the high infection rate among infertile men, there is no increase in DNA fragmentation spermatozoa.
	Luttmer et al. (2016), Golob et al. (2014)	No significant change was found.
	Gizzo et al. (2014)	In vitro evidences showed that HPV infected spermatozoa maintains the ability to fertilize oocytes and to express viral genome in the product of conception.
	Yang et al. (2013)	A loss in motility, vitality and morphology was observed among infected patients.
	Garolla et al. (2013)	Decrease in progressive sperm motility and a higher rate of ASA on the sperm surface
	Garolla et al. (2012)	The virus is present in the head, but is not bound to the acrosome, suggesting that HPV infects spermatozoa by primary binding with syndecan-1
***CT***	Sonnenberg et al. (2013)	Decreased motility, increased non-viable forms of spermatozoa and increased lipid peroxidation of cell membranes due to elevated IgA levels and DNA fragmentation.
	Ahmadi MH et al (2018)	Concentration (milions/ml), total count, progressive motility A, progressive motility B, total progressive motility (A + B + C) and morphology.
	Joki-Korpela (2009), Bezold et al. (2007), Eley et al. (2005),	Bacterial infection may promote deterioration of semen parameters.
	Gallegos (2008)	DNA fragmentation.
	Gunyeli et al. (2011), Hosseinzadeh (2004)	Does not change seminal parameters
